# Effect of Different Levels of Multienzymes on Immune Response, Blood Hematology and Biochemistry, Antioxidants Status and Organs Histology of Broiler Chicks Fed Standard and Low-Density Diets

**DOI:** 10.3389/fvets.2019.00510

**Published:** 2020-02-04

**Authors:** Y. A. Attia, H. Al-Khalaifah, H. S. Abd El-Hamid, M. A. Al-Harthi, A. A. El-shafey

**Affiliations:** ^1^Arid Land Agriculture Department, Faculty of Meteorology, Environment and Arid Land Agriculture, King Abdulaziz University, Jeddah, Saudi Arabia; ^2^Animal and Poultry Production Department, Faculty of Agriculture, Damanhur University, Damanhur, Egypt; ^3^Environment and Life Sciences Research Center, Kuwait Institute for Scientific Research, Kuwait City, Kuwait; ^4^Poultry and Fish Diseases Department, Faculty of Veterinary Medicine, Damanhour University, Damanhur, Egypt

**Keywords:** broilers, nutrient density, multienzymes, immune response, supplementation

## Abstract

This study was executed to investigate the effect of supplementing three multienzyme levels (0, 0. 1, and 0.2%) with two types of diet [standard diet (SD) vs. low-density diet (LDD)] on immune response, blood hematology and biochemistry, antioxidant status, and organ histology of broilers during 1–38 days of age. A total of 216 unsexed 1-day-old Arbor Acres broiler chicks were randomly distributed, on a factorial design (2 × 3), to six treatments each with six replicates. There were six chicks per replicate. Results showed that LDD significantly decreased body weight gain (BWG) of broilers, but did not affect the European Production Efficiency Index (EPEI). Addition of multienzymes at both levels (0.1 and 0.2%) significantly increased BWG and improved EPEI, compared to the control diet. Alanine aminotransferase (ALT), aspirate aminotransferase (AST), malondialdehyde (MDA), lymphocyte, lymphocyte transformation test (LTT), and phagocyte activity (PA) were significantly higher for LDD than the SD, but eosinophil was lower. Supplementation of multienzymes significantly decreased ALT, AST, and MDA, compared to the control group, but increased packed cell volume (PCV), hemoglobin (Hgb), lymphocytes, and monocytes. Immune organs, such as spleen, thymus, and the bursa of Fabricius were significantly increased with multienzyme supplementation. It could be concluded that multienzyme supplementation at either 0.1 or 0.2% to SD or LDD improved EPEI and immune status of broiler chicks.

## Introduction

The cost of poultry feed ingredients represent about 60–70% of the total production cost, and hence, feed formulation is a critical approach in poultry industry. Feed utilization can be met with inclusion of enzymes, antimicrobials, probiotics, or prebiotic or natural products (1–9).

The immunomodulatory effect of supplementing poultry feed with multienzymes has been well-documented in the literature. Hosseindoust et al. (10) concluded that β-mannanase has a potential to improve the gut health of broiler chickens fed with a corn and soybean meal (SBM)–based diet. In the same study, growth performance and the total tract retention of nutrients were also improved. Liu et al. (11) investigated the effect of multienzymes containing phytase, protease, and xylanase at 1,000, 2,000, and 2,000 U/kg of broiler feed, respectively. The authors showed that multienzymes significantly improved feed intake, body weight gain, polymeric Ig receptor (pIgR), secretary IgA (sIgA), and ileal counts of *Lactobacilli* and *Bifidobacteria* and significantly reduced lesions in the intestine, serum a-toxin antibodies, mucin 2 expression, and ileal count of *Clostridium perfringens*. However, the strength of the multienzyme effect depends on the protein content in the diet. The authors pointed that high non-conventional protein in the diet can lead to increased occurrence of subclinical necrotic enteritis, while multienzyme supplementation can reduce this effect in broiler chickens by enhancing the gut immunity (11).

Supplementing poultry feed rations with enzymes can enhance feed utilization and eliminate the negative effect of non-starch polysaccharides (NSPs) in broiler performance (12–14). Multienzyme supplementation, such as amylase, xylanase, and protease is claimed to stimulate disintegration of starch, cell walls, and endogenous proteins, and thus improves energy utilization in corn-SBM and sorghum-SBM diets (15). Attia et al. (16) revealed that supplementing poultry diet with multienzyme enhanced the economic cost of the diets. However, the composition and the type of enzyme mixture determine the effect of the multienzyme supplementation on poultry production performance (17–19).

The use of enzyme complex containing carbohydrases and proteases is claimed to improve utilization of energy, protein, P, and Ca by broilers, laying hens, ducks, and Japanese quail (16, 17, 20, 21). However, there are still conflicting results about the beneficial effect of multienzymes on the poultry performance, and this could be attributed to dietary composition, age, and type of chicks, suggesting the need for further research (17, 22).

The use of low-density diet (LDD) with or without enzyme supplementation in relation to physiological and immunological responses and antioxidant status biochemistry of broilers is a new concept in broilers' nutrition. Most of previous research focused on productive performance and carcass traits as economic criteria (18, 22, 23). In literature, enzyme supplementation, particularly low-energy diets or feedstuffs containing high-fiber and/or antinutritional factors, was found to improve energy use for growth (12–18, 21, 23–26). On the other hand, immunity and antioxidant status and development of the inner organs may need more nutrients than those needs for growth and carcass quality and can perfectly reflect the nutritional status of animals (24, 25). Thus, this study focuses on nutritional intervention between LDD and enzyme supplementation from the point of view of biochemistry, physiological and immunological responses, and antioxidant status and organs histology.

## Materials and Methods

### Chicks, Diet, and Experimental Design

The department committee of Animal and Poultry Production accepts all the procedures done in the current study. These procedures suggest minimal stress to animal to ensure rights and welfare by eliminating harm or suffering to animals according to official decrees of the Ministry of Agriculture in Egypt regarding animal welfare [Decree No. 27 (1967) that enforces the humane treatment of animals generally].

A total of 216 1-day-old Arbor Acres broiler chicks were purchased from Cairo Poultry Company, wing-banded and distributed in random manner, regardless of their sex, with equal initial BW (45.8 ± 1.42) in 36 cages of 6 birds per cage. Each treatment consists of six replicates, with six chicks per replicate.

The experiment was designed with two diets [standard diet (SD) vs. LDD] and three multienzyme levels (unsupplemented, 0.1 and 0.2%) in a factorial design (2 × 3) during 1–38 days of age. Galzym-M are products of Tex Biosciences, UK. Galzym^®^ is a multienzyme containing cellulase 100,000,000 U/kg, 4-β-xylanase 1,500,000 U/kg, lipase 6,500 U/g, α-amylase 250,000 U/kg, protease 40,000 U/kg, pectinase 30,000 U/kg, and sodium benzoate (preservative) 50 mg/kg. The chemical composition of the experimental basal diets fed during the experiment stages is shown in Table 1 (27). The starter diet was fed from hatch until 7 days of age (1 week); a grower diet was fed from 8 to 21 days of age (2–3 weeks); and a finisher diet was fed from 22 to 38 days of age.

**Table 1:** Ingredients and chemical composition of the experimental basal diets fed during the experiment stages.

**Ingredients (g/kg)**	**Standard diets**			**Low density diet**		
	**Starter, 0–7 days of age**	**Grower, 8–21 days of age**	**Finisher, 22–38 days of age**	**Starter, 0–7 days of age**	**Grower, 8–21 days of age**	**Finisher, 22–38 days of age**
Maize	512	517.8	549.2	460.8	466.02	494.28
Wheat bran	0	0	0	100	100	100
Rye	0	50	70	0	45	63
Soybean meal (44% CP)	328	244	284	295.2	219.6	255.6
Dicalcium phosphate	18.00	16.00	10.00	16.20	14.40	9.00
Limestone	10.00	10.00	8.00	9.00	9.00	7.20
NaCl	3.00	4.50	4.50	2.70	4.05	4.05
Full fat soybean meal	100	130	16.0	90	117	14.4
Vit+min premix^a^	3.00	3.00	3.00	2.70	2.70	2.70
L-Lysine	1.00	1.90	2.00	0.90	1.71	1.80
DL-Methionine	2.00	2.50	3.00	1.80	2.25	2.70
Washed building sand	0.30	0.30	0.30	0.27	0.27	0.27
Vegetable oil	22.70	20.00	50.00	20.43	18.00	45.00
Total	1000	1000	1000	1000	1000	1000
**DETERMINED AND CALCULATED AND COMPOSITION (g/kg)**						
CP^b^	227	209	190	220	204	187
ME (MJ/kg)^c^	12.63	12.78	13.37	11.92	12.05	12.58
Calcium^c^	8.58	8.45	6.26	7.86	7.74	5.77
Available phosphorus^c^	4.07	3.78	2.64	3.87	3.60	2.57
Methionine^c^	5.48	5.71	5.94	5.16	5.37	5.57
Methionine + cystine^c^	9.10	9.05	9.02	8.74	8.69	8.67
Lysine^c^	13.18	12.53	11.28	12.50	11.90	10.78
Crude fiber, %^b^	36.1	35.5	33	50.01	46.53	43.5
Ash^b^	51.1	53.5	55	55.2	56	56.2

a *Vit+Min mix. provides per kilogram of the diet: Vit. A, 12,000 IU, vit. E (dl-α-tocopheryl acetate) 20 mg, menadione 2.3 mg, Vit. D3, 2,200 ICU, riboflavin 5.5 mg, calcium pantothenate 12 mg, nicotinic acid 50 mg, choline 250 mg, vit. B_12_ 10 g, vit. B_6_ 3 mg, thiamine 3 mg, folic acid 1 mg, d-biotin 0.05 mg. Trace mineral (mg/kg of diet): Mn 80, Zn 60, Fe 35, Cu 8, selenium 0.1 mg*.

b *Calculated values based on NRC (1994) tables of analyses of feedstuffs*.

c *Determined values*.

### Animal Housing

Chicks were distributed in battery cages in a semi-opened room. The feed rations were fed *ad libitum* and water was freely accessed. A commercial light schedule was followed until the 7th day of 23-h light followed by 20-h light from the 8th day through the rearing phase until the 38th day of age. The outside minimum and maximum temperatures and relative humidity during the rearing phase were averaged as 22.9 and 28.3°C and 60.1 and 62.2%, respectively. The indoor temperature was 33.2, 28.3, and 26.7°C during 1–7, 8–18, and 15–20 days of age, respectively. The vaccination program involves clone 30 on day 8, dual injection of dead Influenza H5 N2 and Newcastle disease virus (NDV) under the skin of the neck on day 10, and clone 30 and Gumboro on day 21.

### Data Collection

The BWG (g/bird) was calculated based on broiler weights (g) at 1, 21, and 38 days of age. Feed conversion rate (FCR) (g feed/g gain) was calculated based on the feed intake (g/bird), and the survival rate (SR, 100 – mortality rate) during 1–21, 22–38, and 1–38 days of age was calculated. European Production Efficiency Index (EPEI) was calculated using the equation of Hubbard Broiler Management Guide (28) as follows:

$$\begin{array}{l} \text{EPEI}=\frac{\text{BW}\left(\text{kg}\right)\;\times \text{ SR}}{\text{PP}\times \text{ FCR}}\times \;100 \end{array}$$

where PP = Production Period (days).

### Collection of Blood Samples

Blood samples (*n* = 6) were randomly withdrawn from each experimental group on day 8 post-vaccination (29 days of age) as well as on day 0 (just before vaccination). Blood samples were withdrawn from the branchial vein by using a vacutainer tube using heparinized and non-heparinized tubes. Serum and plasma were separated by centrifugation at 1,500 × *g* for 15 min. Blood samples used for analyses were collected before the start of all vaccinations (day 0) and after the end of the last vaccination (day 29 of age).

### Hematological Traits

The packed cell volume (PCV %) was determined by centrifuging the blood samples for 20 min at 2,000 × *g* using Wintrobe hematocrit tubes (Jiangdu Sunflower Glass Instrument Factory, Jiangdu, China). The cyanomethemoglobin method was used to determine hemoglobin concentration (Hgb) (29). Red blood cell count was determined using the method of (30), and red cell indices were calculated as described by Jain (31) according to the following equations:

Mean Corpuscular Volume (MCV) (μm^3^) = PCV × 10/RBC's

Mean Corpuscular Hemoglobin (MCH) (Pg) = Hgb × 10/RBC's

Mean Corpuscular Hemoglobin Concentration (MCHC) (g/dl) = Hgb × 100/PCV.

### Immune Indices

Phagocytic activity and index was determined according to Kawahara et al. (32). Phagocytic activity (PA) = percentage of phagocytic cells containing yeast cells. Phagocytic index (PI) = number of yeast cells phagocytized/number of phagocytic cells. This test indicates the activity of the white blood cells that phagocytose harmful foreign particles, bacteria, and dead or dying cells. It is an important immune index, which indicates strongly and reflects the immune status. These phagocytes are mainly monocytes and macrophages, granulocytes, and dendritic cells. The phagocytic index indicates the strength of the phagocytic cell, represented by the number of the ingested bacteria. Phagocytic activity is considered as the first line of defense against antigens and pathogenic agents (33, 34).

Total antibody production specific for NDV vaccine was determined in serum using commercial ELISA kits (35). Antibody response was determined by hemagglutination inhibition (HI) test according to King and Seal (36). The assay is designed to measure IBD antibody bound to influenza antigen-coated plates (37). The Takatsy and Hamar method (38) was used to determine HI against NDV and avian influenza. Lymphocyte transformation test was determined following the method by Balhaa et al. (39). This test measures the T cell proliferation to a drug *in vitro*, from which one can simulate a previous *in vivo* reaction against a drug sensitization. Lymphocyte transformation test has been widely implemented for medical diagnosis of immunodeficiency, pathogenic diseases, and type IV allergy (40, 41). The Rainger and Rowley (42) was used to determine the serum bactericidal activity to *Aeromonas hydrophila* strain, and the results were expressed as survival index. This test is used to measure the bactericidal activity of the serum during antimicrobial treatment against bacteria isolated from the same patient. This test has been widely used in patients with infective endocarditis, osteomyelitis, bacteremia, or other serious bacterial infections (43, 44). The turbidimetric method of Engstad et al. (45) was used to measure the serum lysozyme activity. This test represents the monocyte/macrophage activity and has been widely used to measure the activity of various diseases (46, 47). The reduction in absorbency of 0.001/min refines the result of this test, which is expressed as one unit of lysozyme activity. Lysozyme activity = (A0 – A)/A.

### Biochemical Traits

Commercial kits produced by Diamond diagnostics (23 EL-Montazah St. Heliopolis, Cairo, Egypt, http://www.diamonddiagnostics.com) were used to measure the biochemical traits of the blood. The methods of Armstrong and Carr (48), Doumas et al. (49), and Doumas and Peters (50) were used to measure the total serum protein and albumin concentrations. The method of Cole (51) was used to estimate the globulin concentration through subtraction of albumin concentration from serum total protein. The method of Bossuyt et al. (52) was used to measure the different types of globulin (α-β- and γ-globulin).

The method of Reitman and Frankel (53) was used to determine the activities of alanine aminotransferase (ALT, U/L) and aspartate aminotransferase (AST, U/L). The activities of alkaline phosphatase (ALKP) enzymes were assayed in samples by the method of McComb and Bowers (54). Serum total antioxidant capacity (TAC) and malondialdehyde (MDA) were determined according to Erel (55).

### Histopathological Study

Intestine (i.e., ileum), the bursa of Fabricius, thymus, and spleen specimens were harvested at 38 days of age from six birds from each dietary treatment, *n* = 6, and directly fixed in 10% buffered formalin saline (BFS) for at least 24 h. Conventional paraffin embedding technique was used to process the fixed specimens. This technique involves dehydrating via ascending grades of ethanol, clearing in chloroform, and embedding in paraffin wax at 60C. The resulted paraffin blocks were sliced into slices of 5 μm thick and stained by hematoxyline and eosin (H&E) following the method described by Culling (56).

For morphometrical quantitative measurement of the longitudinal axis of large bursal follicle, five sections per replicate per treatment were examined using Optika imaging analyzer mounted on an Optika binocular microscope. Also, thymus cortical to medullary ratio was qualitatively measured. Spleen was examined to determine lymphoblastic cells hyperplasia presence where (–) means few; (+) means moderate; and (++) means severe.

### Statistical Analysis

The General Linear Model procedure of the Statistical Analysis Software of SAS Institute (57) was applied using two-way factorial design (two types of feeds by three levels of multienzyme) as follows:

yijk = μ + Ai + βj + (Aβ)ij + eijk

Here, μ = general mean, Ai = effect of types of feeds, βj = effect of levels of multienzyme, (Aβ)ij = interaction between feeds and multienzymes, and eijk = random error.

Before analysis, arcsine transformation was done to normalize the data distribution. To confirm the homogeneity (normality test) of the data, the Kolmogorov–Smirnov (K-S) test was used (57). Means are considered different at *P* ≤ 0.05 using Student–Newman–Keuls test.

## Results

### Growth Performance

Table 2 shows the effect of different types of duets and levels of multienzymes on growth of broiler chicks fed with SD and LDD during days 1–38 of age. Results in Table 2 show that the LDD significantly decreased BWG of broilers compared to the SD, but did not affect EPEI during the experimental period. The interaction between the multienzyme level and the type of diet on BWG and EPEI was not significant. Results also indicated that multienzyme supplementation at 0.1 and 0.2% significantly and similarly increased BWG and improved EPEI, as compared to the control diet.

**Table 2:** Effect of different levels of multienzymes on growth of broiler chicks fed with standard and low-density diets during days 1–38 of age.

**Treatment**		**Initial body weight, g**	**Body weight gain (g)/period**			**Production index**
			**1–21 days of age**	**22–38 days of age**	**1–38 days of age**	
**EFFECT OF DIET**						
Standard		45.7	621^a^	1,406	2,045^a^	310
Low density		45.8	580^b^	1,346	1,928^b^	304
SEM		0.315	9.27	11.3	16.0	5.80
**EFFECT OF MULTIENZYMES (%)**						
0		45.9	563^b^	1,266^b^	1,844^b^	281^b^
0.1		45.8	622^a^	1,411^a^	2,036^a^	319^a^
0.2		45.5	616^a^	1,451^a^	2,079^a^	321^a^
SEM		0.386	27.8	34.0	48.1	7.11
**INTERACTION BETWEEN DIET AND MULTIENZYME SUPPLEMENTATION (%)**						
Standard	0	45.4	614^a^	1,300	1,937	288
	0.1	46.3	628^a^	1,431	2,071	320
	0.2	45.5	621^a^	1,487	2,126	323
Low density	0	46.5	512^b^	1,233	1,750	274
	0.1	45.4	616^a^	1,391	2,001	318
	0.2	45.6	612^a^	1,415	2,031	319
SEM		0.546	32.4	39.7	56.2	10.0
***P*****-VALUE**						
Diet		0.804	0.004	0.139	0.016	0.426
Multienzymes		0.740	0.001	0.002	0.001	0.001
Interaction		0.209	0.01	0.935	0.554	0.811

a,b,c *Means within a column with different superscripts are significantly different based on SNK test*.

*SEM, standard error of the mean*.

### Hematological Traits of Blood

Tables 3, 4 show the effect of experimental diets on hematological traits of blood and differential white blood cells, respectively. PCV, Hgb, RBCs, MCV, MCH, MCHC, WBCs, and different types of WBC were not affected by the type of diet. Lymphocytes had significantly higher LDD while the opposite trend was shown in the case of eosinophils (Table 3). Supplementation of multienzymes significantly increased Hgb, PCV (Table 3), lymphocyte, and monocyte (Table 4) compared to control diet. There was a significant interaction between type of diet and multienzyme concentrations on Hgb, PCV (Table 3), and WBCs (Table 4), showing that multienzyme increased Hgb and PCV of the SD in a stepwise manner, but did not affect the LDD, showing that the impact of multienzyme on Hgb and PCV depends on the type of diet. The lymphocyte response to multienzyme dose was in a stepwise manner (Table 4).

**Table 3:** Effect of different concentrations of enzyme cocktail on blood hematological traits of broiler chicks fed with standard and low-density diets.

**Treatment**		**PCV (ml/100 ml)**	**Hemoglobin (g/100 ml)**	**RBC's (10^12^/L)**	**MCV (μm^3^/RBC)^1^**	**MCH (pg)^2^**	**MCHC (%)^3^**
**EFFECT OF DIET %**							
Standard		30.0	10.0	1.66	182	60.8	33.3
Low density		30.0	10.1	1.62	188	63.3	33.7
SEM		0.427	0.17	0.025	4.35	1.47	0.45
**EFFECT OF MULTIENZYMES (%)**							
0		28.3^b^	9.43^b^	1.60	180	59.7	33.2
0.1		30.4^a^	10.1^a^	1.66	185	61.9	33.4
0.2		31.4^a^	10.6^a^	1.66	191	64.4	33.8
SEM		0.273	0.208	0.031	5.33	1.81	0.552
**INTERACTION BETWEEN DIET AND MULTIENZYME SUPPLEMENTATION (%)**							
Standard	0	27.0^c^	8.75^c^	1.58	172	55.7	32.4
	0.1	30.7^b^	10.37^a^^,^^b^	1.71	182	61.1	33.7
	0.2	32.5^a^	11.0^a^	1.68	194	65.5	33.8
Low density	0	29.7^b^	10.1^b^	1.61	188	63.8	34
	0.1	30.1^b^	10.0^b^	1.61	188	62.7	33.2
	0.2	30.3^b^	10.2^b^	1.63	187	63.3	33.8
SEM		0.739	0.295	0.044	7.54	2.091	0.781
***P*****-VALUE: THE** ***P*****-VALUE FOR THE INTERACTION WAS ADDED**							
Diet		0.990	0.732	0.267	0.399	0.235	0.574
Enzyme		0.001	0.001	0.293	0.370	0.205	0.760
Interaction		0.007	0.002	0.390	0.322	0.138	0.381

a,b,c *Means within a column with different superscripts are significantly different based on SNK test*.

1 MCV, Mean corpuscular volume;

2 MCH, Mean corpuscular hemoglobin;

3 *MCHC, Mean corpuscular hemoglobin concentration. Number of observations were six per interaction cell. SEM, standard error of the mean*.

**Table 4:** Effect of different concentrations of enzyme cocktail on white blood cell and its fractions of broiler chicks fed with standard and low-density diet.

**Treatment**		**WBCs (10^9^/L)**	**Lymphocytes (%)**	**Monocytes (%)**	**Basophils (%)**	**Eosinophils (%)**	**Heterophylis (%)**	**H/L ratio**
**EFFECT OF DIET**								
Standard		22.6	41.0^b^	10.6	0.54	10.0^a^	23.5	0.577
Low density		22.8	42.0^a^	10.7	0.50	9.0^b^	23.8	0.572
SEM		0.19	0.32	0.186	0.12	0.171	0.286	0.082
**EFFECT OF MULTIENZYME SUPPLEMENTATION (%)**								
0		22.8	40.0^c^	9.68^b^	0.56	9.87	23.1	0.583
0.1		22.6	41.7^b^	11.3^a^	0.43	9.37	24.1	0.583
0.2		22.8	42.9^a^	11.1^a^	0.56	9.43	23.8	0.558
SEM		0.232	0.392	0.228	0.147	0.209	0.35	0.101
**INTERACTION BETWEEN DIET AND MULTIENZYME SUPPLEMENTATION (%)**								
Standard	0	21.7^c^	39.7	9.25	0.625	10.6	23.0	0.585
	0.1	23.3^a^^,^^b^	40.8	11.3	0.501	9.87	23.7	0.583
	0.2	22.7^b^	42.6	11.3	0.500	9.75	24.0	0.563
Low density	0	23.8^a^	40.3	10.1	0.500	9.12	23.2	0.581
	0.1	21.8^c^	42.6	11.2	0.375	8.87	24.6	0.582
	0.2	22.8^b^	43.2	10.8	0.625	9.12	23.7	0.552
SEM		0.329	0.555	0.323	0.208	0.296	0.496	0.142
***P*****-VALUE**								
Diet		0.358	0.034	0.754	0.808	0.001	0.476	0.644
Enzyme		0.807	0.001	0.001	0.788	0.199	0.102	0.141
Interaction		0.001	0.512	0.103	0.788	0.345	0.530	0.935

a,b,c *Means within a column with different superscripts are significantly different based on SNK test*.

*H/L ratio, Heterophil number/lymphocyte number as indication of stress; number of observations were six per interaction cell. SEM, standard error of the mean*.

There was a significant interaction between the type of diet and the multienzyme level only on WBCs, showing that EC supplementation to SD significantly increased WBCs but decreased LDD, showing that the impact of multienzymes on WBCs depends on the type of diet.

### Biochemical Constituents of Blood

Data for biochemical constituents of blood are shown in Tables 5, 6. The ALT, AST, and MAD were significantly higher in LDD than in SD, but TAC/MDA (antioxidants balance) was lower (Table 5). The total protein, albumin, α-, β-, and γ-globulin, globulin, and globulin/albumin ratio were not significantly influenced by the type of diet (Table 6).

**Table 5:** Effect of different concentrations of enzyme on liver enzymes, blood serum malondialdehyde, and total antioxidant capacity of broiler chicks fed with standard and low-density diet.

**Treatment**		**AST (U/L)**	**ALT (U/L)**	**AST/ALT ratio**	**Alkaline phosphatase (U/L)**	**MDA (mMol/dl)**	**TAC (mMol/dl)**	**TAC/MDA ratio**
**EFFECT OF DIET**								
Standard		63.5^b^	54.8^b^	1.15	11.0	1.26^b^	425	337.3^a^
Low density		65.2^a^	56.7^a^	1.15	11.3	1.46^a^	424	290.4^b^
SEM		0.255	0.443	0.0086	0.224	0.037	2.03	1.56
**EFFECT OF MULTIENZYME SUPPLEMENTATION (%)**								
0		66.0^a^	58.0^a^	1.14	11.5	1.57^a^	436^a^	277.7^b^
0.1		63.1^b^	54.8^b^	1.15	10.6	1.26^b^	417^b^	331.0^a^
0.2		63.9^b^	54.5^b^	1.17	11.4	1.25^b^	419^b^	335.2^a^
SEM		0.313	0.543	0.01	0.275	0.045	2.48	1.91
**INTERACTION BETWEEN DIET AND MULTIENZYME SUPPLEMENTATION (%)**								
Standard	0	65.5	57.1	1.14	11.6	1.41	443^a^	314.2^b^
	0.1	61.7	53.1	1.16	10.5	1.17	414^c^	353.8^a^
	0.2	63.2	54.3	1.16	11.1	1.21	418^c^	345.5^a^
Low density	0	66.6	58.8	1.13	11.3	1.73	430^b^	248.6^c^
	0.1	64.5	56.6	1.14	10.8	1.36	420^b^^c^	308.8^b^
	0.2	64.6	54.6	1.18	11.7	1.31	421^b^^c^	321.4^b^
SEM		0.443	0.768	0.015	0.388	0.064	3.52	2.70
***P*****-VALUE**								
Diet		0.001	0.006	0.679	0.436	0.005	0.656	0.003
Enzyme		0.001	0.001	0.100	0.080	0.001	0.001	0.001
Interaction		0.156	0.120	0.349	0.517	0.193	0.022	0.036

a,b,c *Means within a column with different superscripts are significantly different based on SNK test*.

*ALT, Alanine aminotransferase; AST, aspartate aminotransferase; AST/ALT, aspartate aminotransferase to alanine aminotransferase ratio; MDA, malondialdehyde. Number of observations was 12 per interaction cell. SEM, standard error of the mean*.

**Table 6:** Effect of different concentrations of enzyme cocktail on biochemical constituents of blood serum of broiler chicks fed with standard and low-density diet.

**Treatment**		**Serum biochemical constituents (g/100 ml)**						
		**Total protein**	**Albumin**	**Globulin**	**α-Globulin**	**β-Globulin**	**γ-Globulin**	**GAR**
**EFFECT OF DIET**								
Standard		4.91	2.70	2.21	0.951	0.708	0.549	0.825
Low density		4.85	2.73	2.12	0.879	0.709	0.533	0.779
SEM		0.075	0.088	0.064	0.041	0.021	0.038	0.044
**EFFECT OF MULTIENZYME SUPPLEMENTATION (%)**								
0		4.73	2.64	2.09	0.837	0.651^b^	0.600	0.797
0.1		4.86	2.70	2.16	0.906	0.750^a^	0.506	0.809
0.2		5.06	2.81	2.24	1.003	0.712^a^^b^	0.519	0.802
SEM		0.092	0.108	0.078	0.05	0.026	0.047	0.054
**INTERACTION BETWEEN DIET AND MULTIENZYME SUPPLEMENTATION (%)**								
Standard	0	4.78	2.63	2.15	0.837	0.637	0.675	0.821
	0.1	4.86	2.75	2.11	0.887	0.762	0.462	0.783
	0.2	5.10	2.72	2.37	1.130	0.725	0.512	0.872
Low density	0	4.68	2.65	2.03	0.837	0.675	0.525	0.773
	0.1	4.86	2.65	2.21	0.925	0.737	0.550	0.834
	0.2	5.02	2.91	2.11	0.875	0.712	0.525	0.731
SEM		0.13	0.153	0.111	0.07	0.037	0.67	0.076
***P*****-VALUE**								
Diet		0.588	0.792	0.320	0.205	0.990	0.763	0.369
Enzyme		0.055	0.515	0.412	0.071	0.050	0.327	0.992
Interaction		0.924	0.645	0.275	0.084	0.680	0.208	0.453

a,b,c *Means within a column with different superscripts are significantly different based on SNK test*.

*GAR, Globulin to albumin ratio. Number of observations were 12 per interaction cell. SEM, standard error of the mean*.

Supplementation of multienzymes at 0.1 and 0.2% significantly decreased the ALT, AST, MDA, and TAC compared to the control group (Table 5), but increased antioxidant balance (TAC/MDA). Liver enzyme ratio (ALT/AST) and alkaline phosphatase were not significantly affected by multienzyme supplementation. There was a significant effect of multienzymes on β-globulin showing greater values of chicks on diet with 0.2 and 0.1% multienzymes than those on the control diet without multienzyme supplementation (Table 6). The multienzyme effect on the total protein was moderate (*P* = 0.057).

There was no significant influence of the interaction between multienzyme supplementation and type of diet on blood MDA, protein, and liver functions as reflected by blood enzymes (ALT, AST, ALT/AST, and alkaline phosphatase). However, a significant effect of the interaction was observed in the TAC and antioxidant balance (TAC/MDA). The results indicated an increase in the TAC/MDA ratio of broilers supplemented with enzymes of SD and LDD.

### Lymph Organs and Antibody Titer

Table 7 shows the effect of different concentrations of multienzymes on immune organs and antibody titer of broilers fed with SD and LDD. The type of diet had no significant effect on lymph organs, such as spleen, thymus, and the bursa of Fabricius and HI for NDV. However, HI for AI was significantly higher in LDD than that in SD. These organs were also significantly higher for broilers on diet supplemented with either 0.1 or 0.2% multienzymes than those on diet without EC supplementations.

**Table 7:** Effect of different concentrations of enzyme cocktail on immune organs and antibody titer of broiler chicks fed with standard and low-density diet.

**Treatment**		**Spleen**		**Thymus**		**Bursa of fabricius**		**HI (log2)**	
		**Weight (g)**	**Percentage (%)**	**Weight (g)**	**Percentage (%)**	**Weight (g)**	**Percentage (%)**	**NDV**	**AI**
**EFFECT OF DIET**									
Standard		2.19	0.12	7.72	0.46	2.92	0.19	3.22	0.58^b^
Low density		2.13	0.13	8.08	0.52	3.14	0.21	3.55	1.62^a^
SEM		0.092	0.004	0.201	0.017	0.122	0.008	2.77	0.197
**EFFECT OF MULTIENZYME SUPPLEMENTATION (%)**									
0		1.69^b^	0.110^b^	6.37^b^	0.425^b^	2.32^b^	0.167^b^	1.12^b^	0.125^b^
0.1		2.52^a^	0.147^a^	8.42^a^	0.503^a^	3.26^a^	0.208^a^	4.12^a^	1.81^a^
0.2		2.27^a^	0.139^a^	8.91^a^	0.556^a^	3.51^a^	0.230^a^	4.91^a^	1.37^a^
SEM		0.113	0.005	0.247	0.020	0.349	0.01	0.339	0.241
**INTERACTION BETWEEN DIET AND MULTIENZYME SUPPLEMENTATION (%)**									
Standard	0	1.97^c^	0.112	8.19	0.366	2.51^c^	0.162	1.16	0.25^c^
	0.1	2.34^b^	0.135	8.20	0.481	2.91^b^, ^c^	0.187	3.41	1.12^b^
	0.2	2.26^b^, ^c^	0.136	8.76	0.539	3.35^a^, ^b^	0.224	5.08	0.37^c^
Low density	0	1.40^d^	0.108	6.55	0.484	2.14^c^	0.171	1.08	0.00^c^
	0.1	2.71^a^	0.159	8.64	0.524	3.61^a^	0.228	4.83	2.50^a^
	0.2	2.29^b^, ^c^	0.142	9.07	0.573	3.61^a^	0.237	4.75	2.37^a^
SEM		0.160	0.008	0.349	0.029	0.212	0.015	0.480	0.371
***P*****-VALUE**									
Diet		0.654	0.252	0.207	0.010	0.224	0.094	0.400	0.004
Enzyme		0.001	0.002	0.001	0.002	0.001	0.005	0.001	0.001
Interaction		0.017	0.258	0.982	0.300	0.047	0.513	0.152	0.030

a,b,c,d *Means within a column with different superscripts are significantly different based on SNK test*.

*Number of observations were six per interaction cell. SEM, standard error of the mean*.

There were significant interactions between multienzyme supplementation and the type of diet on the absolute weight of spleen and the bursa of Fabricius and HI for AI. The results showed that multienzyme supplementation significantly increased the weight of spleen and the bursa of Fabricius and HI for AI. However, multienzyme supplementation at 0.1% significantly increased the weight of spleen compared to the unsupplemented control, whereas 0.2% multienzyme increased the absolute weight of the bursa of Fabricius.

### Immune Indices

Table 8 shows the effect of the different experimental diets on the immune indices. There was a significant effect of the type of diet on LTT, BACT, and PA, showing the enhancing effect of LDD on the immune response (Table 8).

**Table 8:** Effect of different concentrations of enzyme cocktail on immune index of broiler chicks fed with standard and low-density diet.

**Treatment**		**LTT (%)**	**BACT (%)**	**LYS (%)**	**PI**	**PA (%)**
**EFFECT OF DIET**						
Standard		23.2^b^	42.2^b^	0.080	1.58	17.2^b^
Low density		26.3^a^	43.9^a^	0.120	1.56	19.0^a^
SEM		0.596	0.571	0.018	0.026	0.318
**EFFECT OF MULTIENZYME SUPPLEMENTATION (%)**						
0		24.8	43.9	0.080	1.46^b^	17.5
0.1		24.8	42.6	0.130	1.58^a^	18.1
0.2		24.6	42.7	0.090	1.68^a^	18.6
SEM		0.697	0.699	0.022	0.032	0.389
**INTERACTION BETWEEN DIET AND MULTIENZYME SUPPLEMENTATION (%)**						
Standard	0	23.6	43.2	0.071	1.45	16.0
	0.1	22.8	41.7	0.087	1.58	17.2
	0.2	23.2	41.8	0.086	1.72	18.5
Low density	0	26.1	44.6	0.091	1.47	19
	0.1	26.8	43.6	0.176	1.58	19.1
	0.2	26.0	43.6	0.097	1.63	18.8
SEM		9.86	9.89	0.031	0.046	0.551
***P*-VALUE**						
Diet		0.001	0.046	0.130	0.589	0.004
Enzyme		0.958	0.373	0.253	0.002	0.110
Interaction		0.720	0.966	0.415	0.459	0.070

a,b,c *Means within a column with different superscripts are significantly different based on SNK test*.

*LTT, Lymphocyte Transformation Test; BACT, Bactericidal Activity; LYS, Lysozyme Activity; TAC, Total Antioxidant Capacity; PI, Phagocytic Index; PA, Phagocytic Activity. Number of observations were six per interaction cell. SEM, standard error of the mean*.

Supplementation of multienzymes significantly increased PI compared to the control group and had no effect on the other immune indices, such as LTT, BACT, LYS, and PA.

There was no significant influence of the interaction between the dose of multienzymes and the type of diet on LTT, BACT, LYS, PI, and PA.

### Histology Study

**Figure 1:**
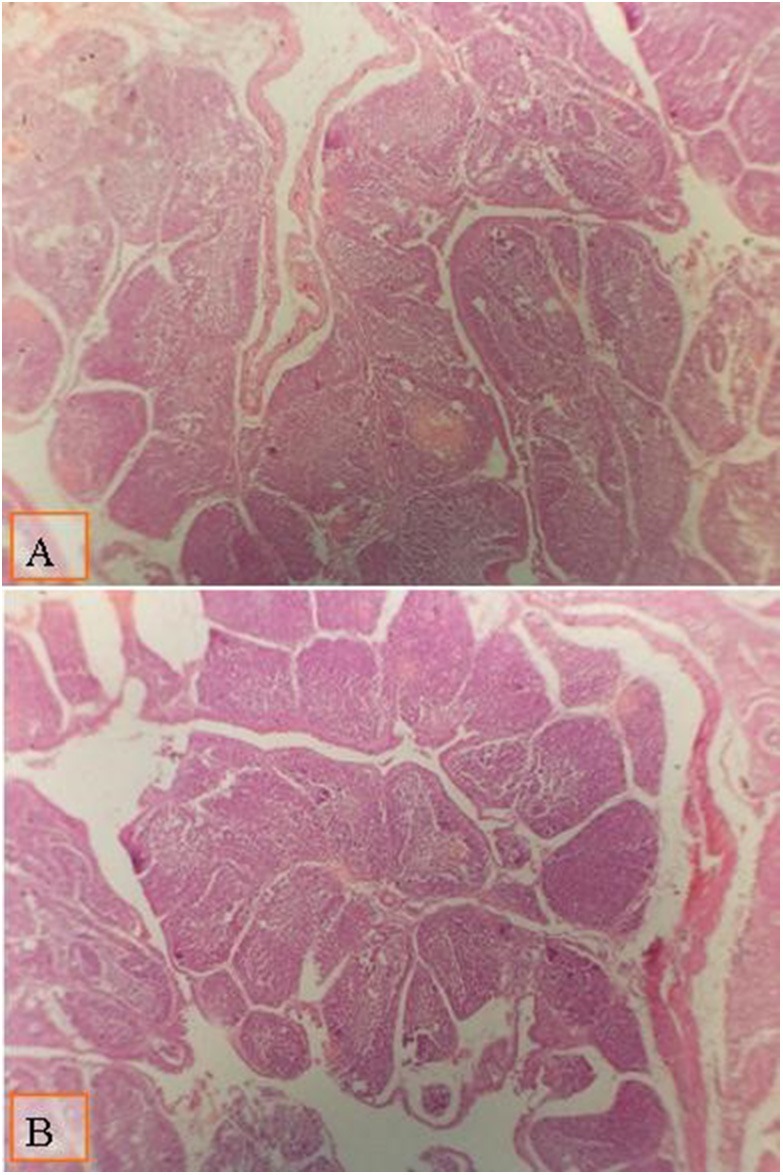
Micrograph of the bursa of Fabricius of broiler at day 28 of age stained with H&E (×40) to compare the follicle diameter in different groups; the distance between two follicular polar as shown all groups by lines: **(A)** broiler fed with a standard diet supplemented with 0.1% ml enzyme cocktail; **(B)** broiler fed with a diet supplemented with 0.2% of multienzyme. Moderate increase in the follicular diameter was noticed in broiler fed with diet supplemented with 0.1% of multienzyme **(A)**.

**Figure 2:**
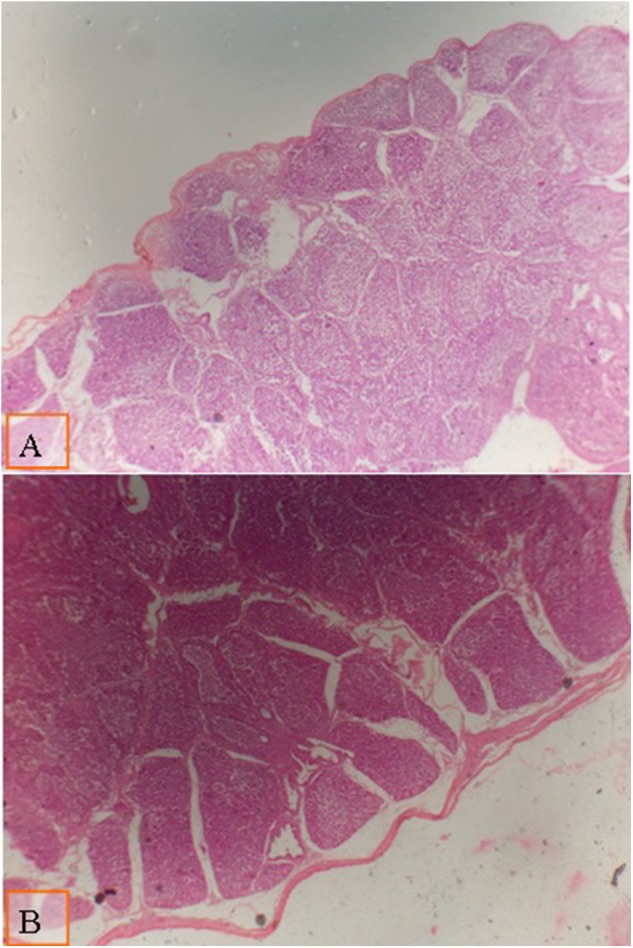
Micrograph of the bursa of Fabricius of broiler at day 28 of age stained with H&E (×40) to compare the follicle diameter in different groups; the distance between two follicular polar as shown all groups by lines: **(A)** broiler fed with low density diet supplemented with 0.1% of multienzyme; **(B)** broiler fed with a diet supplemented with 0.2% of multienzyme. Moderate increase in the follicular diameter was noticed in broiler fed with a diet supplemented with 0.1% of multienzyme **(A)**.

The effect of the type of diet and multienzyme supplementation on the morphology of the intestine and the bursa of Fabricius is shown in Table 9. The type of diet had no significant effect on the diameter of the large follicle of the bursa of Fabricius. There was a significant increase in the diameter of the large follicle of bursa of Fabricius (Figures 1, 2; Table 9) due to supplementation of 0.1% multienzyme, as compared to the other multienzyme concentrations. The increase in the bursa of Fabricius amounted to 16.6%, respectively. There was also insignificant increase due to supplementation of 0.2% multienzymes.

**Table 9:** Effect of type of diet and enzyme cocktail supplementation of morphology of the intestinal and *Fabricius bursa*.

**Treatment**		**Length of intestinal villi (μm)**	**L. axis of large follicle of *Fabricius bursa* (μm)**
**EFFECT OF DIET**			
Standard		235	166
Low density		250	174
SEM		5.76	4.39
**EFFECT OF MULTIENZYME SUPPLEMENTATION (%)**			
0		215^b^	157^b^
0.1		285^a^	183^a^
0.2		226^b^	169^a^^,^ ^b^
SEM		7.05	5.38
**INTERACTION BETWEEN DIET AND MULTIENZYME**			
**SUPPLEMENTATION (%)**			
Standard	0	211	157
	0.1	269	175
	0.2	223	163
Low density	0	219	157
	0.1	301	191
	0.2	228	174
SEM		9.98	7.6
***P*****-VALUE**			
Diet		0.078	0.166
Enzyme		0.001	0.008
Interaction		0.348	0.537

a,b,c *Means within a column with different superscripts are significantly different based on SNK test. SEM, standard error of the mean. The number of replicates was six per treatment and the number of sections/slide was five per replicate per treatment*.

**Figure 3:**
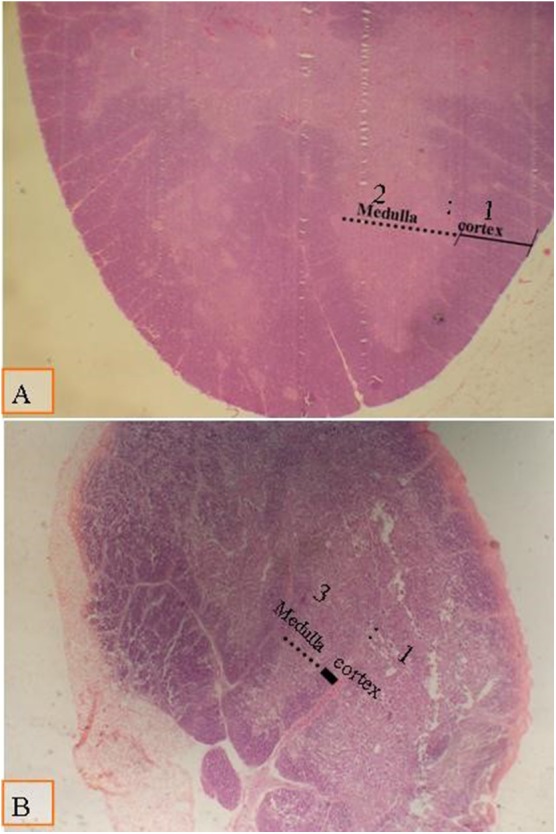
Micrograph of the thymus stained with H&E (×40) to compare the thymic cortical/medullary ratio: **(A)** the cortic/medullary ratio is 2:1 as shown in all groups at day 28 of age; **(B)** the cortic/medullary ration is 3:1 as shown in all groups at day 38 of age, which associated with physiological aged thymic atrophy.

**Figure 4:**
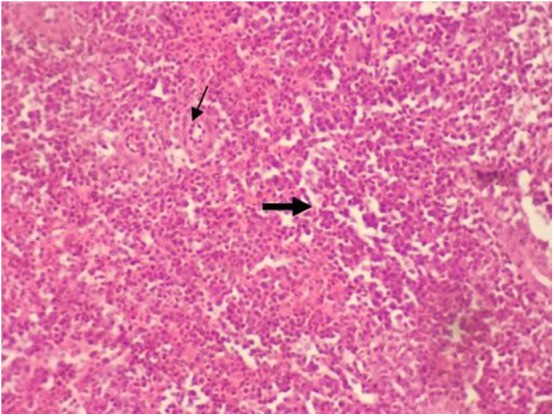
Micrograph of the spleen stained with H&E (×40) of control group shown with normal splenic histology featuring splenic arteriole (thin arrow) with white and red pulp (thick arrow). All groups showed the normal splenic histology as control.

There were no significant changes in spleen and thymus due to type of diet and multienzyme supplementation and their interaction (Figures 3 and 4). However, cortical to medullary ratio in thymus was decreased with increasing age of chicks. With time, the thymus began to atrophy and the decrease the cortical to medullary ratio became physiological.

## Discussion

This work was executed to study the effect of supplementing three multienzyme levels (0, 0.1, and 0.2%) with two types of diet (SD vs. LDD) on immune response, blood hematology and biochemistry, antioxidant status, and organ histology of broilers during 1–38 days of age. The trend in using LDD in feeding broiler chicks has been recently discussed and may be a possible approach to decrease growth pressure on the skeletal system of birds, and reduce feed cost and environmental pollution (23, 24, 58). Data showed that inclusion of wheat bran in broiler diets at 10% induced a significant growth depression in BWG and FCR only during the early growth period, reaching 6.61 and 6.74%, respectively, which persisted in only BWG during the whole period, reaching 5.6%. This showed that LDD did not provide adequate nutrients for muscle growth during different experiment periods. However, broiler tolerance to dietary composition increased with increasing age of chicks (17, 59). The current results are in agreement with those reported by Al-Harthi (60) and Attia (16, 17). The enhanced BW and BWG as a result of multienzyme supplementation may be due to the increased nutrient availability and absorption as a result of increased digestibility of the ingested diets, as suggested by Choct (22), Attia et al. (18), and El-Kelawy (61). However, the effects of multienzyme depend on the composition of the diet and the enzyme type (17, 62). It was previously reported that multienzyme supplementation increased BWG and FCR while reducing feed intake. Multienzyme significantly increased the growth during 7–21 days of age, and this is explained by the existence of amylase and NSPs degrading enzymes (23, 60, 63, 64). Adding exogenous enzymes that hydrolyze the NSP of vegetable ingredients in the diets for monogastric enhances the energy availability and use of nutrients, and thus enhances feed conversion ratio (65). The increase in the release of nutrient due to enzyme supplementation resulted in higher nutrient available for absorption, as demonstrated by the increase in intestinal villi length, and thus for biochemical reaction in favor of anabolic reaction and muscle buildup (18) and for immune function as well (21, 25).

The use of LDD enhanced LTT and PA, indicating higher immunity. This enhancement in immunity could be attributed to redistribution of nutrients toward immunity rather than growth. Growth was decreased due to feeding LDD and thus the availability of nutrients increased for physiological response, immunity, and antioxidant utilization for eliminating free radical resulting from both non-enzymatic and enzymatic reactions due to decreasing nutrient demands for growth. For example, the TAC/MDA ratio (antioxidant balance) was significantly increased due to feeding enzyme-supplemented diet. This increase reflected the significant decrease in the antioxidants (TAC) for use in protection of cell from free radicals (7, 25).

Inclusion of wheat bran in the diet and particularly polysaccharides has been reported to be antioxidants, immunomodulators, anti-inflammatory, antitussive, anticancerous, and antimutagenic (1, 66–68). In addition, wheat bran and arabinoxylans have been shown to improve phagocytosis of macrophages in animal models. They also stimulate humoral response in chickens. It improved production of total IgS, IgG, and IgM anti-SRBC antibody titers on days 7 and 14 PPI and PSI of SRBCs, relative to the standard (69, 70). Administration of arabinoxylans in the diet significantly increased anti-SRBC antibody response in arabinoxylans-administered chickens and suggested increased humoral immune response, which may be attributed to an improved animal's potential to encounter disease agents (71). In addition, wheat bran is rich in phytase enzyme, which can improve nutrient availability by chickens, such as protein, energy, and minerals (17, 72).

Interestingly, the current results showed that EC improved the EPEI of broiler chicks. This result is in line with those shown by Al-Harthi (60) and Attia (16, 17). The improved EPPI due to EC supplementation was concurred with greater villi length for the group on 0.1% multienzyme supplementation; this is in line with the findings of Choct (22) and Attia et al. (18). However, the effect of multienzyme depends on diet composition and the type of enzyme (17, 62). The effect of enzyme supplementation on animal performance could be a result of the effect of amylase and NSPs degrading enzymes (23, 60, 63, 64). The addition of exogenous enzymes that hydrolyze the NSP of vegetable ingredients in the feeds for monogastric enhances the availability of energy and use of nutrients and thus enhances FCR (65). In addition, Gao et al. (73) reported that xylanase supplementation of wheat-based diets for cockerels significantly improved the production of serum antibody response to NDV as a mean of humoral response. On the contrary, Basmacioglu Malayoglu et al. (74) revealed that NSP-degrading enzyme supplementation had no significant effect on immune response represented by titers of IgG and IgM. Also, Khaksar et al. (75) found that the relative weight of immune organs (thymus, spleen, and the bursa of Fabricius) was not influenced by enzyme supplementation.

In addition, dietary composition intervention with enzymes was recently studied by Head et al. (76) who suggested that if broilers are supplemented with flaxseed, the nutrient digestibility and the availability of n-3 FA will be limited due to the presence of non-starch polysaccharides (NSP), by affecting the genetic process during lipid metabolism in the liver. The results revealed that enzyme addition to diets supplemented with 10% flaxseed decreased arachidonic acid and total long chain n-6 FA. Dietary flaxseed and enzyme treatments upregulated PPARα target genes *CPT1A* and *ACOX1* while reducing the expression of *de novo* FA synthesis-related genes (76). In addition, Seidavi et al. (77) demonstrated that supplementing the diet with probiotic/enzyme mixture had no effect on the humoral response against AI and NDV on day 42. There was a significant difference of IgG after the second challenge with SRBC's (*P* = 0.003). A lower body weight of the birds was found in most of the treated groups, relative to the standard (*P* = 0.031). The absolute and relative weight of the spleen was significantly different compared to the control group (*P* = 0.003 and *P* = 0.001, respectively). The weights of the thymus and the bursa of Fabricius were not different (77). Further study was conducted by Abdel-Hafeez et al. (78) who found that using potato peels at 15% and sugar beet pulp at 7.5% decreased the body weight. Enzyme inclusion significantly improved the body weight in potato peels and sugar beet pulp. The total cholesterol and low-density lipoprotein cholesterol serum levels were reduced in all experimental groups. Also, the carcass fat content was reduced using the potato peels and sugar beet pulp, with or without enzyme (78). This indicates that enzymes supplementation affects blood biochemistry of broilers (61) and thus meat quality improved because of decreasing carcass fat (18).

These results suggest that it is possible to dilute nutrient profiles of broiler diets during the growing and finishing phases without negative effect on EPEI and EC while improving immune response and economic efficiency. These results are in agreement with those reported by Abudabos (23).

The use of LDD in the current study did not negatively affect villi length. The relationship between gut morphology and the type of diet was reported in the literature; the use of cereal with a high NSP level may increase the size of the gastrointestinal tract (72, 79). A significant positive correlation between arabinoxylan level in wheat and the relative weights of the duodenum, jejunum, and ileum has been reported (80). It is known that diet makeup may induce microscopic changes in the intestinal mucosa, and it is possible that dietary NSP levels may also affect the morphology of the gastrointestinal tract (81). Iji (82) reported that the crypt depth of both the jejunum and ileum was improved due to supplementation with guar gum and xanthan gum, indicating that NSP may stimulate gastrointestinal tract cell turnover. The increased crypt depth indicated increased villus cell stimulation and thus an increase in nutrients being absorbed and thus being utilized by the gastrointestinal tract. This indicates that cereal type affects the gastrointestinal tract size and the morphology of the intestine (83).

It is well-known that increasing the cortex in relation to the medulla ratio increased the number of T-lymphoblasts and increased the T-lymphocyte, which reflects on stimulating cellular immunity (84–86).

A recent study by Woyengo et al. (87) showed that using amylase, NSPase, or a combination of amylase and NSPase in the phytase-supplemented basal diet further improved (*P* < 0.05) ileum digestibility to 63.4, 69.9, and 67.3%, respectively. However, the dietary nitrogen corrected apparent metabolizable energy value was not affected by the addition of phytase, amylase, or a combination of amylase and NSPase. Woyengo et al. (87) concluded that the addition of amylase and NSPase to broiler phytase-supplemented diets is beneficial to improve apparent metabolizable energy corrected by nitrogen. In another study by Sateri et al. (88), they showed no significant effects of olive meal and enzymes on growth performance and on cecum microflora. Antibody titers against infectious bronchitis virus (IBV) and Gumboro disease were higher in birds fed with 4% olive meal. Supplementing the diet with enzymes did not affect the aforementioned parameters (88).

The present results indicate that EC supplementations improved the immunity of broiler chicks as measured by organ changes. This was concurred with an increasing diameter of large follicle of the bursa of Fabricius for the group supplemented with 0.1% multienzymes. The increase in the follicular diameter of the bursa of Fabricius indicates an increase in the number of B-lymphoblasts that leads to the formulation of B-lymphocytes that internally form antibodies. This reflects stimulation of the humoral immunity due to increased nutrient available for antibody formation (89, 90). In addition, the phagocytic index was similarly increased with supplementation of 0.1 and 0.2% multienzyme concentrations. The mechanism(s) by which wheat bran and/or enzyme supplementation positively affected chick's immunity may involve an improvement in gut health due to the decrease in harmful microbiota in the hind gut, increase in the intestinal villi length, and/or increase in the nutrient available for immune function (21, 24, 25, 73, 89, 90). In this regard, Gao et al. (73) reported that feeding cockerels on wheat-based diets supplemented with xylanase significantly enhances serum antibodies to NDV, indicating that enzyme supplementations enhance the humoral response due to increased nutrient utilization for immune function. But in another study, there was no significant effect on humoral response (IgG and IgM) with NSP-degrading enzyme supplementation (74). Also, Khaksar et al. (75) found that the relative weight of immune organs (thymus, spleen, and the bursa of Fabricius) was not influenced by enzyme supplementation. This contraindication in published literature indicated that immunity of birds is a complex concept that is affected by diet composition, age, and stress of animals (24, 25, 84).

In conclusion, multienzyme supplementation at either 0.1 or 0.2% to SD or LDD improved the production index while enhancing the immune response of broilers during age of 1–38 days.

## Data Availability Statement

The datasets generated for this study are available on request to the corresponding author.

## Ethics Statement

The animal study was reviewed and approved by the Department Committee of Animal and Poultry Production, official decrees of the Ministry of Agriculture in Egypt relevant to animal welfare are No. 27 (1967).

## Author Contributions

All authors listed have made a substantial, direct and intellectual contribution to the work, and approved it for publication.

### Conflict of Interest

The authors declare that the research was conducted in the absence of any commercial or financial relationships that could be construed as a potential conflict of interest. The handling editor declared a past co-authorship with one of the authors YA.
